# Machine learning for the identification of respiratory viral attachment machinery from sequences data

**DOI:** 10.1371/journal.pone.0281642

**Published:** 2023-03-02

**Authors:** Kenji C. Walker, Maïa Shwarts, Stepan Demidikin, Arijit Chakravarty, Diane Joseph-McCarthy

**Affiliations:** 1 Department of Biomedical Engineering, Boston University, Boston, Massachusetts, United States of America; 2 Fractal Therapeutics, Cambridge, Massachusetts, United States of America; California State University Fresno, UNITED STATES

## Abstract

At the outset of an emergent viral respiratory pandemic, sequence data is among the first molecular information available. As viral attachment machinery is a key target for therapeutic and prophylactic interventions, rapid identification of viral “spike” proteins from sequence can significantly accelerate the development of medical countermeasures. For six families of respiratory viruses, covering the vast majority of airborne and droplet-transmitted diseases, host cell entry is mediated by the binding of viral surface glycoproteins that interact with a host cell receptor. In this report it is shown that sequence data for an unknown virus belonging to one of the six families above provides sufficient information to identify the protein(s) responsible for viral attachment. Random forest models that take as input a set of respiratory viral sequences can classify the protein as “spike” vs. non-spike based on predicted secondary structure elements alone (with 97.3% correctly classified) or in combination with N-glycosylation related features (with 97.0% correctly classified). Models were validated through 10-fold cross-validation, bootstrapping on a class-balanced set, and an out-of-sample extra-familial validation set. Surprisingly, we showed that secondary structural elements and N-glycosylation features were sufficient for model generation. The ability to rapidly identify viral attachment machinery directly from sequence data holds the potential to accelerate the design of medical countermeasures for future pandemics. Furthermore, this approach may be extendable for the identification of other potential viral targets and for viral sequence annotation in general in the future.

## Introduction

The COVID-19 pandemic has underscored the importance of an effective response for emerging viral pathogens that is focused on the rapid deployment of molecular testing and medical countermeasures. Our experiences with the current pandemic have highlighted the vulnerability of the global healthcare infrastructure to respiratory pathogens that, like SARS-CoV-2, are capable of long-range airborne spread via aerosolized particles [1]. In contrast to other pathogens, the window for effective intervention to avert a pandemic resulting from a newly emergent respiratory virus may be very short. Thus, the speed with which molecular diagnostics, therapeutics, and vaccines can be deployed are critical determinants of our ability to contain an outbreak.

The viral attachment machinery (the set of proteins responsible for host cell attachment and cell entry) has served as a historically important focus for the development of molecular tests (for example for influenza [2] and SARS-CoV-2 [3, 4]) as well as medical countermeasures such as vaccines [5–7]. Thus, the accurate and efficient identification of the viral attachment machinery is a critical first step in the design and deployment of biomedical countermeasures. It had been observed for coronaviruses in 2012 (pre-COVID-19) that the tertiary structure of the spike protein is not conserved but that the secondary structure topology is conserved [8]. It was subsequently also noted that the pattern of N-linked glycosylation is highly conserved and may play a role in immune evasion [9].

The identification of viral attachment machinery from sequence can be thought of as a special case of the larger problem of automated function prediction (AFP) of novel proteins, which is a mature field (see [10–13] for reviews). A number of groups have used approaches for AFP that leverage structure-based homology, focusing either on the full three-dimensional (3D) protein structure, or on the identification of 3D structural motifs (see, for example, [14–17]). However, 3D structure alone is often insufficient for functional annotation, as proteins possessing similar global structures can perform very different biological functions (for example, [18]). Computational structural alignment methods, although first pioneered in the 1960s, typically have accuracies on the order of ~90% [19] but at least in the case of coronaviruses as described above the 3D structure is not conserved. Furthermore, 3D structural motifs for viral attachment proteins are often optimized specifically for enzymes and are not readily able to identify viral attachment machinery. As an alternative, AFP from DNA sequences relies on sequence homology [20–22], or the identification of sequence motifs [23, 24]. A potential weakness of this approach is that novel viruses with low sequence homology to pre-existing pathogens may prove less tractable to homology-based approaches. As a further consideration, during the early days of an emerging pandemic, steps such as multiple sequence alignment, phylogeny reconstruction and 3D structure prediction can add weeks to the timeline for response. An accurate ML model may be able to pinpoint the target within seconds.

With respect to preparedness for potential future pandemics, tools that can aid in the rapid deployment of therapeutic and vaccine countermeasures are clearly needed. Specifically, for viral pathogens originating from the most prevalent respiratory virus families, which are key pathogens of concern, intervening at the localized emergence stage may prevent the transition to a full-blown pandemic. Based on the earlier cited observations, we hypothesized it may be possible to develop a machine learning (ML) model based on predicted secondary structure elements and N-glycosylation features alone capable of identifying viral attachment machinery (the “spike” protein or its equivalent) from an unknown respiratory virus sequence. More generally, we also sought to gain a further understanding of the structural features that may distinguish viral attachment machinery proteins with a view toward elucidation of key structure-function relationships.

## Methods

### Virus families, viral sequences, and “spike” proteins

**Fig 1:**
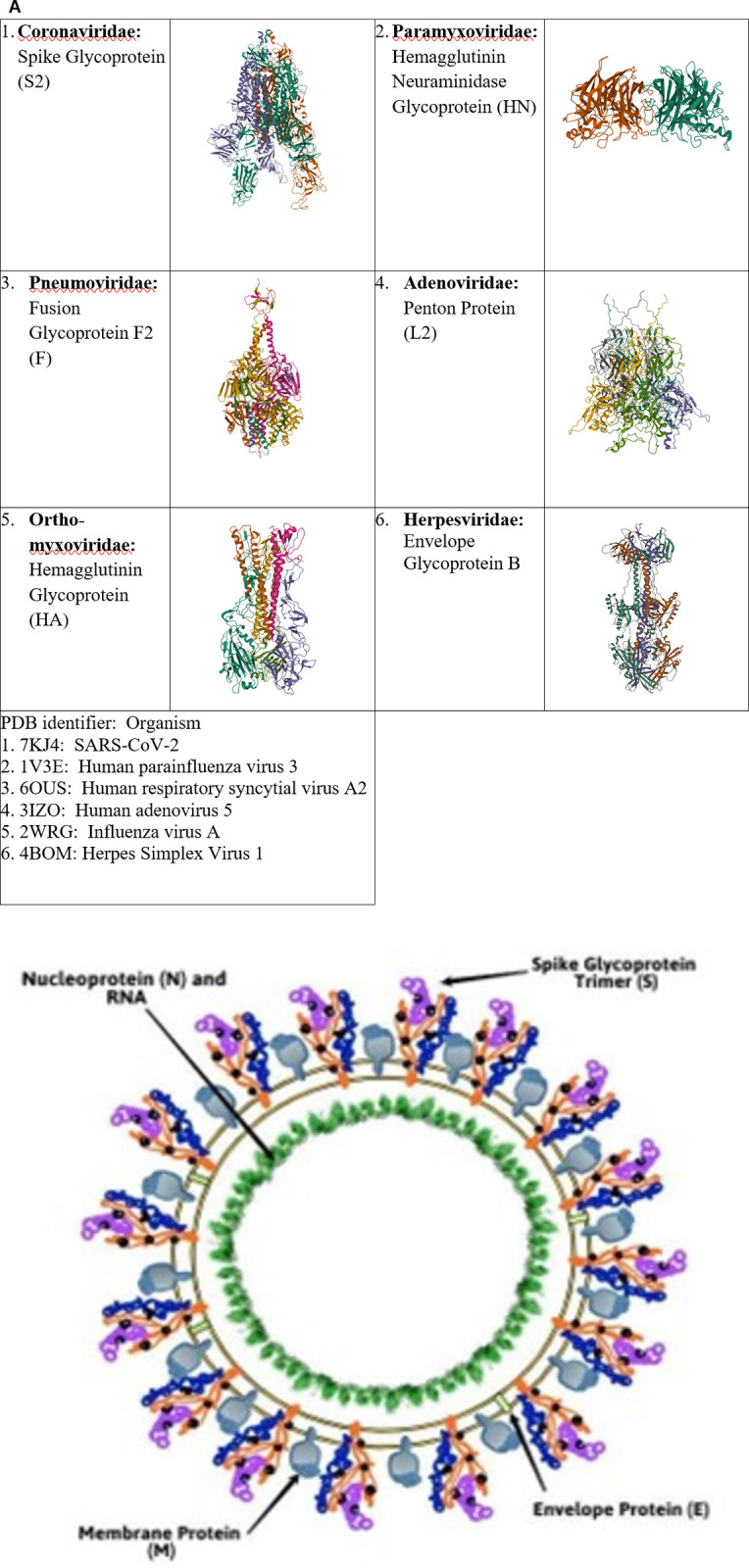
Five families of respiratory viruses and their “spike” proteins. In (A) the identity and representative structure of the “spike” protein (gene name given in parentheses) is shown for each of the virus families studied. PDB identifiers for structures 1–6 are also listed with the corresponding virus indicated. Shown in (B) is a schematic of the coronavirus SARS-CoV-2 structure indicating the prominence of the spike.

Across all sets (feature selection, training, extra-familial validation), six families of respiratory viruses were included in this study: Coronaviridae, Paramyxoviridae, Pneumoviridae, Adenoviridae, Orthomyxoviridae, and Herpesviridae. Each of the viruses within these families has a protein responsible for viral attachment and host cell entry, which will be referred to herein as the “spike” protein (see Fig 1A). For Coronaviruses, it is the Spike S Glycoprotein which is aptly named because it projects from the surface of the virion (Fig 1B) as do the other “spike” proteins. Note that for Influenza Virus A within the Orthomyxoviridae family, we selected Hemagglutinin as the equivalent of the “spike” over Neuraminidase as the latter primarily prevents virion aggregation and as such serves more as a helper protein to the role of the former in determining cell entry [25].

A total of 50 viral sequences (ranging from 4 to 12 for each virus family) encoding 360 proteins were utilized (see Table 1 for a list of sequences). Specifically, in the feature selection set we included 7 Coronaviridae sequences representing 7 viruses; in the training set, we included 7 different Coronaviridae sequences representing 7 viruses, 4 Paramyxoviridae sequences representing 4 viruses, 12 Pneumoviridae sequences representing 2 viruses, 8 Adenoviridae sequences representing 1 virus, and 8 Orthomyxoviridae sequences representing 1 virus. Finally, for the extra-familial validation set, we included 4 Herpesviridae sequences representing 4 viruses. See Table 2 for the number of “spike” vs. non-spike proteins for each virus family.

**Table 1:** NCBI reference respiratory virus sequences used in model development.

Feature Selection Set Sequences			
Virus Family	Virus^a^	Strain	Sequence Identifier^b^
Coronaviridae	SARS-CoV-2 [26]	Wuhan-Hu-1	NC_045512.2
Coronaviridae	SARS-CoV-1 [27]	Tor2	NC_004718.3
Coronaviridae	MERS [28]	HCoV-EMC/2012	NC_019843.3
Coronaviridae	hCoV-OC43 [29]	ATCC VR-759	NC_006213.1
Coronaviridae	hCoV-HKU1 [30]	HKU1	NC_006577.2
Coronaviridae	hCoV-NL63 [30]	Amsterdam I	NC_005831.2
Coronaviridae	hCoV-229E [30]	299E	NC_002645.1
**Training Set Sequences**			
Virus Family	Virusa	Strain	Sequence Identifierb
Coronaviridae	Bat Coronavirus	1A	NC_010437.1
Coronaviridae	Turkey Coronavirus	MG10	NC_010800.1
Coronaviridae	Bulbul Coronavirus	HKU11-934	NC_011547.1
Coronaviridae	Betacoronavirus HKU24	HKU24	NC_026011.1
Coronaviridae	Bat Coronavirus	CMR704-P12	NC_048212.1
Coronaviridae	Canada Goose Coronavirus	Cambridge_Bay_2017	NC_046965.1
Coronaviridae	Thrush Coronavirus	HKU12-600	NC_011549.1
Paramyxoviridae	HPIV 1 [31]	Washington 1964	NC_003461.1
Paramyxoviridae	HPIV 2 [31]	VIROAF10	KM190939.1
Paramyxoviridae	HPIV 3 [31]	GP	NC_001796.2
Paramyxoviridae	HPIV 4a [31]	M-25	NC_021928.1
Pneumoviridae	HRSV [32]	Subgroup A	NC_038235.1
Pneumoviridae	HRSV	CA-17	LC385004.1
Pneumoviridae	HRSV	CA-15	LC385003.1
Pneumoviridae	HRSV	KW-15	LC385002.1
Pneumoviridae	HMPV [33]	PER/FPP00726/2011/A	KJ627437.1
Pneumoviridae	HMPV	Isolate 00–1	NC_039199.1
Pneumoviridae	HMPV	PER/IPE00957/2012/A	KJ627433.1
Pneumoviridae	HMPV	Seattle/USA/SC0380/2019	MN306028.1
Pneumoviridae	HMPV	01/KEN/2015	MK588634.1
Pneumoviridae	HMPV	USA/NM013/2016	KY474543.1
Pneumoviridae	HMPV	BuenosAires/ARG/001/2016	MG773272.1
Pneumoviridae	HMPV	AUS/183219938/2004/B	KF530178.1
Adenoviridae	HAdV [34]	Type 2	J01917.1
Adenoviridae	HAdV [35]	Type 3	DQ086466.1
Adenoviridae	HAdV [36]	Type 4	KF006344.1
Adenoviridae	HAdV [37]	Type 5	AC_000008.1
Adenoviridae	HAdV [35]	Type 7	AC_000018.1
Adenoviridae	HAdV [38]	Type 14	AY803294.1
Adenoviridae	HAdV [34]	Type 35	AC_000019.1
Adenoviridae	HAdV [39]	Type 55	MG905110.1
Orthomyxoviridae	Influenza Virus A [40]	A/chicken/Morocco/SF5/2016 (H9N2)	LT598501.1 LT598506.1 LT598511.1 LT598516.1 LT598521.1 LT598526.1 LT598531.1 LT598536.1
Orthomyxoviridae	Influenza Virus A	A/California/07/2009 (H1N1)	YP_009118626.1 YP_009118628.1 CY121687.1 KU933483.1 CY121682.1 CY121684 KU933488.1 CY121683.1
Orthomyxoviridae	Influenza Virus A	A/Berlin/3/1964 (H2N2)	ACD85187.1 ACD85195.1 ACD85197.1 ACD85194.1 ACD85190.1 ACD85192.1 ACD85188.1 ACD85191.1
Orthomyxoviridae	Influenza Virus A	A/Shanghai/02/2013 (H7N9)	NC_026425.1 NC_026423.1 NC_026422.1 NC_026424.1 NC_026429.1 NC_026428.1 NC_026427.1 NC_026426.1
Orthomyxoviridae	Influenza Virus A	A/ruddy turnstone/Delaware Bay/262/2006 (H7N3)	ACO95657.1 ACO95665.1 ACO95667.1 ACO95664.1 ACO95660.1 ACO95662.1 ACO95658.1 ACO95661.1
Orthomyxoviridae	Influenza Virus A	A/Chicken/Hong Kong/715.5/01 (H5N1)	AF509025.1 AF509178.2 AF509152.2 AF509204.2 AF509100.2 AF509075.1 AF509049.1 AF509126.2
Orthomyxoviridae	Influenza Virus A	A/swine/France/IIIeetVilaine-0346/2011 (H1N2)	KC894804.1 KR701484.1 KR701483.1 KR701485.1 KC894807.1 KR701488.1 KR701487.1 KR701486.1
Orthomyxoviridae	Influenza Virus A	A/swine/Texas/4199-2/1998(H3N2))	AEK70342.1 AAD51248.1 AEK70339.1 AEK70341.1 AEK70343.1 AEK70344.1 AEK70345.1 AEK70347.1
**Extra-Familial Set Sequences**			
Virus Family	Virusa	Strain	Sequence Identifierb
Herpesviridae	Herpes Simplex Virus 1 [41]	17	NC_001806.2
Herpesviridae	Herpes Simplex Virus 2	HG52	NC_001798.2
Herpesviridae	Porcine Cytomegalovirus	BJ09	NC_022233.1
Herpesviridae	Cynomolgus Macaque Cytomegalovirus	Ottawa	NC_016154.1

^a^ MERS = Middle East Respiratory Syndrome, HPIV = human parainfluenza virus, HRSV = human respiratory syncytial virus, HMPV = human metapneumovirus, HAdV = human adenovirus; references indicate that the virus is responsible for respiratory disease.

**Table 2:** Summary of respiratory virus families representation in model datasets.

Viral Family	Viral Species Represented	Labeled Spike Proteins	Labeled Non-spike Proteins
Coronaviridae	14	14	51
Paramyxoviridae	4	4	27
Pneumoviridae	2	12	101
Adenoviridae	1	8	55
Orthomyxovride	1	8	56
Herpesviridae	4	4	20

### Prediction of secondary structural elements

The Jpred4 [42] secondary structure prediction server was used to predict structural elements for each viral sequence in the dataset. Jpred4 is a server that hosts Jnet, a neural network secondary structure prediction algorithm trained with different representations of multiple sequence alignment profiles for the same sequences [43]. Each residue in a protein sequence is designated as H (helical), E (extended sheet), or other. Since Jpred4 predicts secondary structure on protein sequences up to 800 amino acids in length, a fully automated script (S1 Fig) was written to break protein sequences into 800 residue segments and subsequently concatenated the results. For each protein, the script calculates the protein length, the percentage of residues in the protein predicted to be helical (%helix), and the percentage predicted in an extended sheet (%sheet). It then identifies the longest contiguous stretch of helix and extended sheet in the protein and calculates %longest helix, and %longest sheet, where %longest helix (sheet) is the length of the longest helical (extended sheet) stretch in the protein divided by the length of the protein. Finally, %helix, %sheet, %longest helix, and %longest sheet is output.

### Prediction of N-glycosylation sites

For the sequences described above, N-glycosylation sites were predicted for each protein using NetNGlyc [44, 45]. The NetNGlyc method uses artificial neural networks to predicts N-Glycosylation sites in proteins through analysis of the sequence context of Asn-Xaa-Ser/Thr sequons. FASTA format protein sequences were entered on the NetNGlyc 1.0 Server (https://services.healthtech.dtu.dk). Asparagines with overall positive score, denoted by ‘+’, ‘++’, ‘+++’ and ‘++++’ (each counted in their respective category), where ‘++++’ indicates a prediction with highest confidence based on a combination of overall potential score and jury agreement amongst the nine neural networks utilized, were predicted to be glycosylated. The total number of glycosylation sites per protein (total N-sites) was the sum of the number of residues scored ‘+’ or higher. The density was the total sites divided by the number of residues in the protein (as reported by NetNGlyc).

### Amino acid composition

Protein sequences were obtained from nucleic acid sequences with Bioinformatics Toolbox in MATLAB version 2019b (MathWorks, 2021, Natick, MA, USA), and a letter frequency counter code was used to obtain the occurrence of each amino acid (AA) for each protein. The individual occurrences were divided by the corresponding protein amino acid length and multiplied by 100, giving %AA composition.

### Statistical test of association

Two-tailed t-tests for independent samples were performed using XLSTAT v22.2.3 (Addinsoft, 2020 New York, USA) to assess the association of various features with spike vs. non-spike protein status. Features that showed a statistically significant association (*p*-value ≤ 0.05) between spike and non-spike groups and thereby rejected the null hypothesis were considered for inclusion in the ML models.

### Input vectors for ML models

Feature vectors were generated for each of the 360 protein sequences and allocated to their appropriate dataset (described above and see Table 1). For each protein, the following features were calculated as described above: total N-sites, density, %M, %N, %S, %sheet, %helix, %longest sheet, and %longest helix. The designation of spike or non-spike was also included.

### Random forest model development

Weka, an open-source software workbench for ML and data analysis [46], was utilized to develop Random Forest classifiers derived from the dataset described above. Random Forest was utilized because is a supervised ensemble learning method that generates a set of uncorrelated decision trees maximizing the separation of the classes that are sought to be discriminated, leading to models robust to overfitting [47, 48]. Data were converted into ARFF format (uploaded as Supporting Information) for input to the Weka Explorer version 3.8.4 to generate specific Random Forest models (see S1 Table). For each Random Forest model, a class-balanced score was also generated. The statistical significance of each model result relative to the class-balanced score was assessed by performing a two-sided Fisher’s exact test with an alpha cutoff of 0.05 [49]. For all Random Forest models, default hyperparameters were used—100 trees using 2 predictors with an unlimited tree depth. Furthermore, for all models, assessment was performed with stratified 10-fold cross validation while an additional extra-familial validation set was used to assess cross-family models. Model performance was evaluated by %correctly classified and AUC metrics were generated from the Receiver Operating Characteristic (ROC) curve to indicate model performance across classification thresholds. A 95% confidence interval for the AUC was calculated using the Real Statistics package for Excel to estimate the true AUC performance.

### Bootstrapping

**Fig 2:**
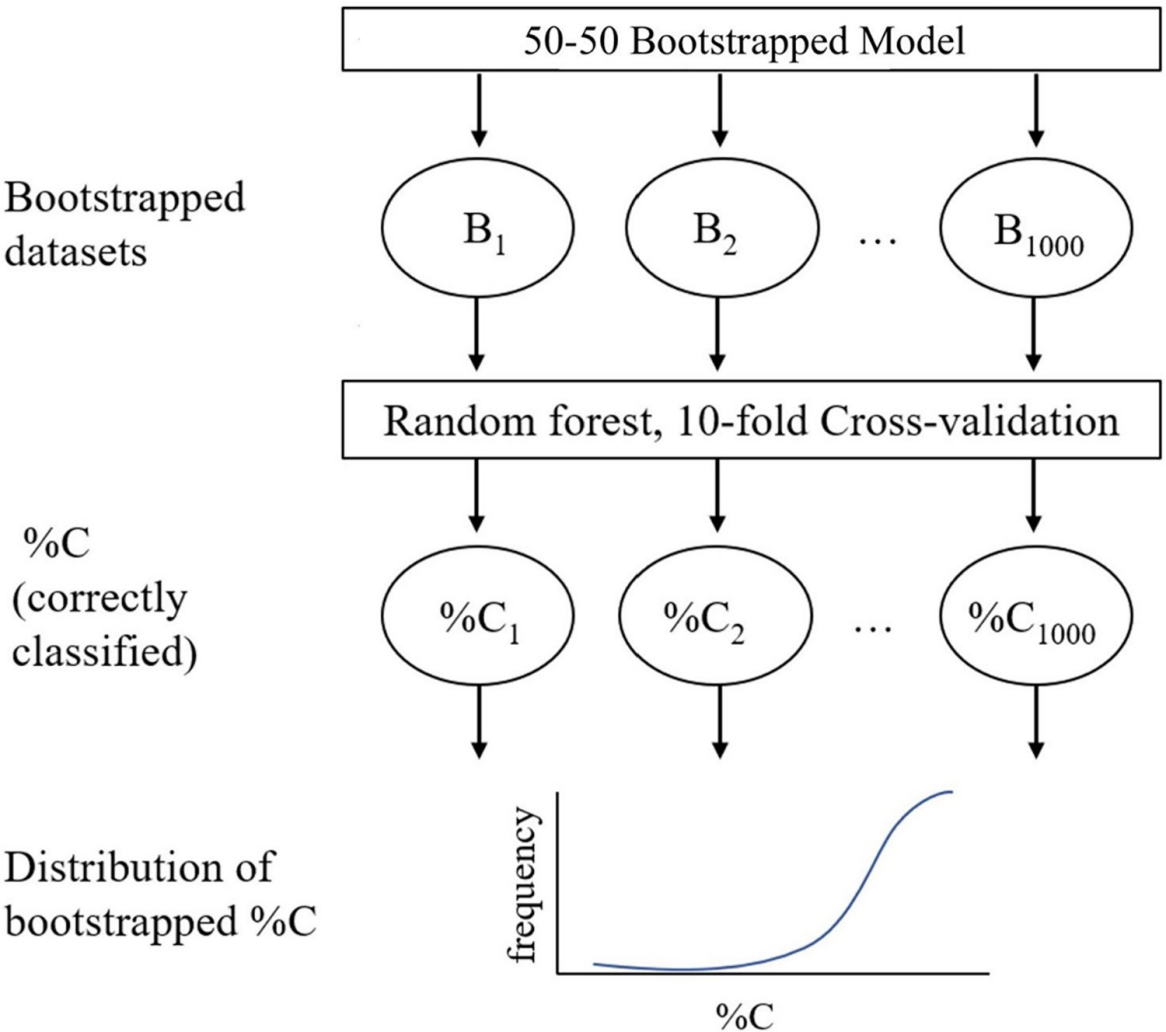
Schematic of bootstrapping process for cross validation of selected models. In this case, each of the 1000 bootstrapped datasets contains feature vectors for 337 protein sequences.

One thousand 50–50 balanced bootstrapping datasets were generated from the training set using the Weka resample filter biased towards a uniform class as depicted in Fig 2. Specifically, 50% of the dataset, 168 proteins, was for proteins designated as spike, and the other 50% were for those designated as non-spike, while retaining the same number of total instances.

## Results

**Fig 3:**
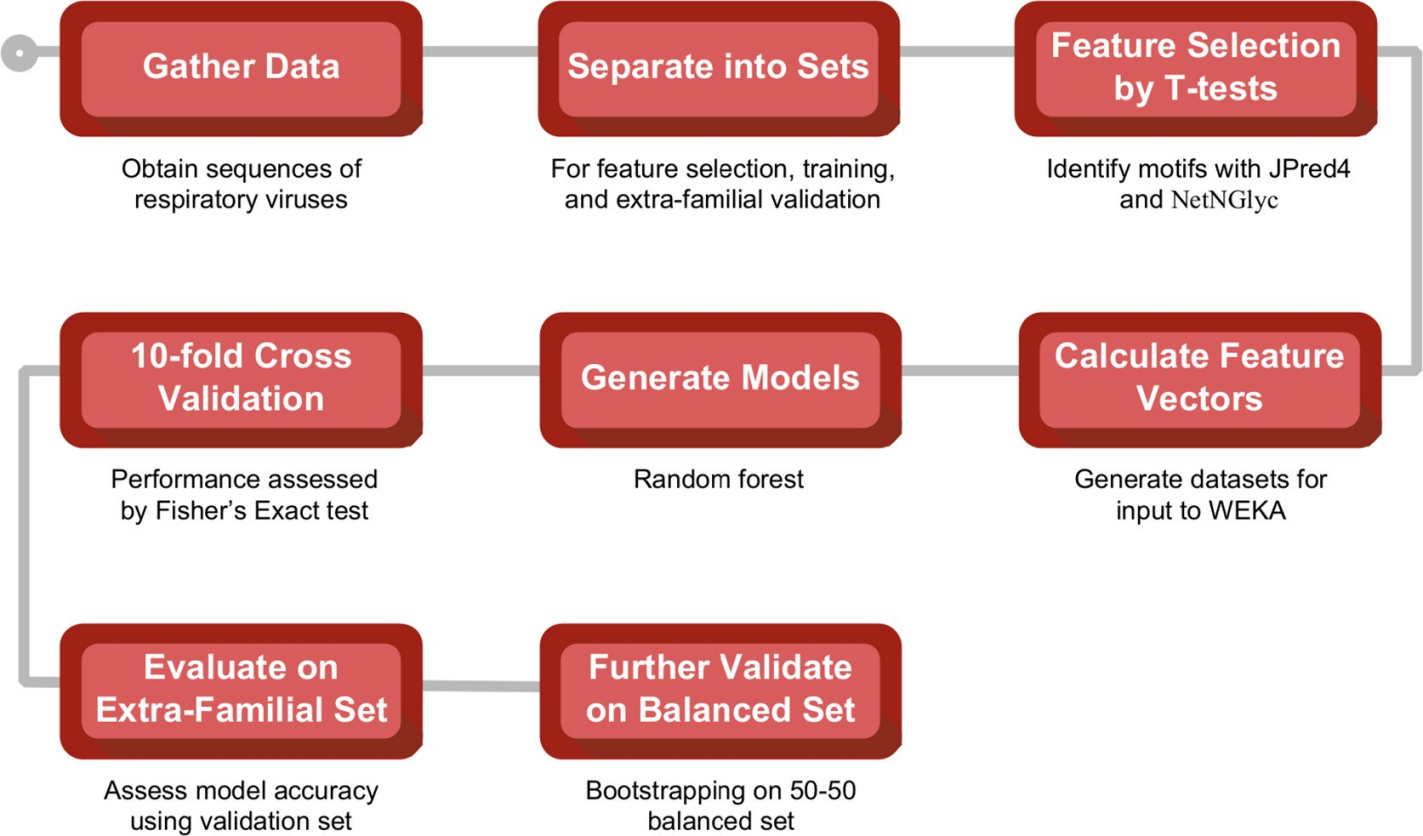
Overall model development workflow. The procedure for the development of ML models to differentiate Spike from non-Spike in a sequence.

To examine the feasibility of using a machine learning model trained on viral sequences to predict “spike” vs. non-spike, a data set was assembled consisting of, in total, 360 protein sequences for 50 respiratory viruses from six virus families, with each protein labeled as “spike” (viral attachment machinery) or non-spike. and then allocated to the appropriate subset—feature selection, training, or extra-familial validation (Table 1). Next, using the feature selection set, the associations between various features and the classification of “spike” vs. non-spike for coronaviruses were examined to look for signals indicating that certain feature types may help to differentiate “spike” vs. non-spike. The overall workflow for model development is outlined in Fig 3.

For the coronavirus sequences in the feature selection set, two-tailed t-tests were performed looking at the association of %helix, %sheet, %longest sheet, %longest helix, respectively, with spike vs. non-spike status (see Table 3 for p-values, which have not been corrected for multiple comparisons). A statistically significant association was observed for %sheet, whereas none was for %helix, %longest helix, and %longest sheet. The %longest helix was examined because when predicted secondary structure topology was examined across the SARS-CoV-2 sequence (NC_045512.2) the spike region appeared to have more longer helical segments than the other regions of the sequence; %longest sheet was added for completeness.

**Table 3:** Results of t-test for spike and non-spike distributions of features used.

Features	P-value
%sheet	0.001
%helix	0.087
%longest sheet	0.208
%longest helix	0.083
Total N-sites	<0.0001
N-sites Density	0.010
%M	0.032
%N	0.008
%S	0.030

Also, for the feature selection coronavirus sequences, t-tests were performed examining the correlation of total N-sites and density, respectively, for spike vs. non-spike (Table 3). A significant statistical difference was found for the total N-sites and density. The %AA was also examined over the coronaviruses dataset to determine if there were significant differences in amino acid composition for spike vs. non-spike. Of the 20%AAs, a significant difference was observed for %N, %S, and %M (refer to Table 3).

**Fig 4:**
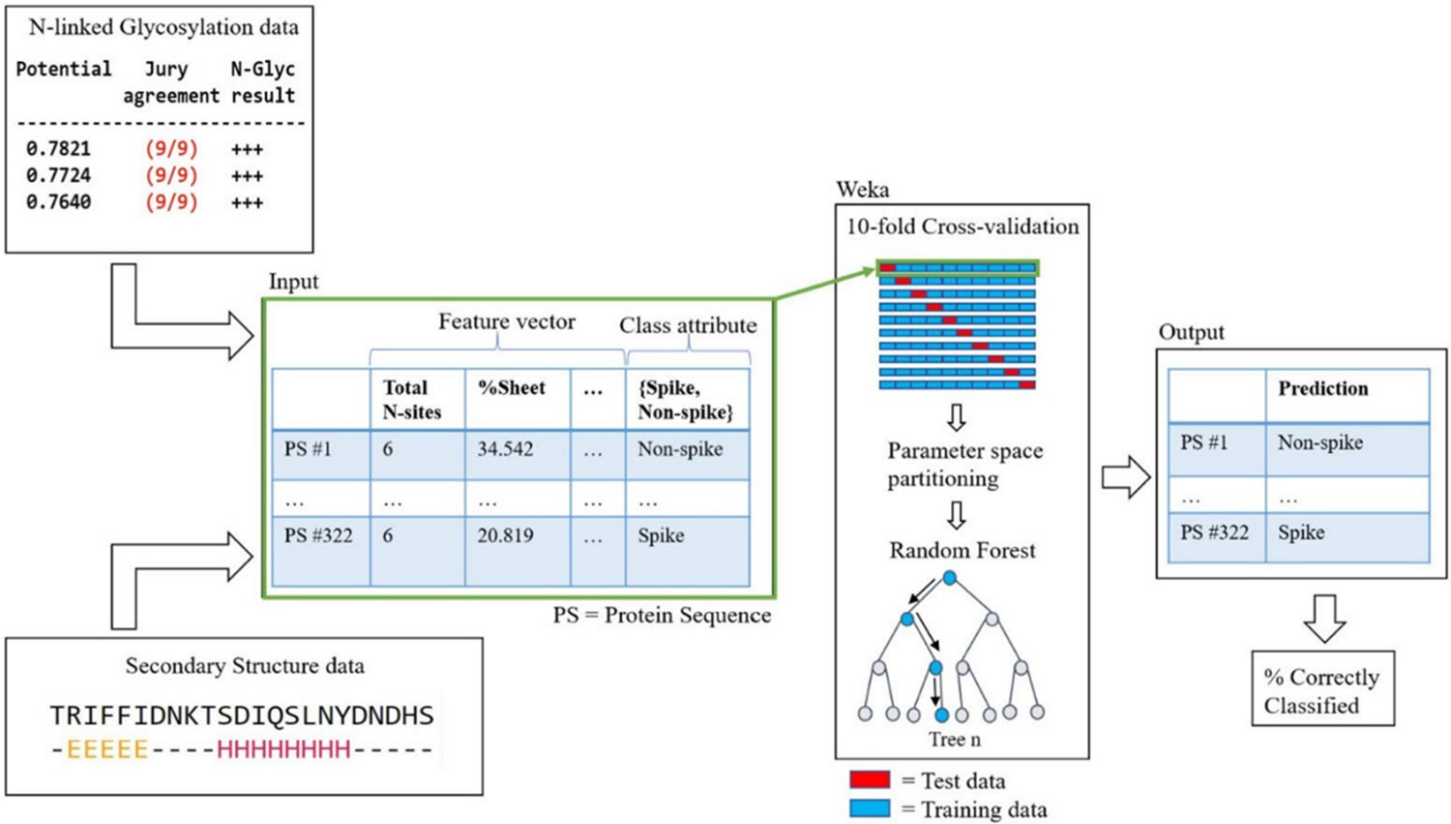
Random forest inputs, cross validation, and outputs. Data was input for 360 protein sequences.

Based on these preliminary findings, we developed Random Forest machine learning classifiers with a feature vector that consisted of glycosylation, amino acid composition, and secondary structure element related features (see Fig 4). To place these results in context, we compared classifier accuracy in each case to the class-balanced score for the same dataset. The class-balanced score is equivalent to the performance of a classifier which simply predicts the majority class, non-spike in this context, providing a benchmark for classification performance. We also performed a test of association between the class-balanced score and model accuracy using a two-tailed Fisher’s exact test.

Our first set of Random Forest models were developed based on the coronavirus sequences in the training set and validated using 10-fold cross validation (see S1 Table). All models classified the proteins correctly 98.6% of the time with a class-balanced score of 86.4% and a *p*-value of 0.028 (two-tailed Fisher’s Exact Test). A comparison of these five models suggests that only total N-sites, N-site density, and secondary structure features may contribute significantly to the models. Next, the secondary structure feature vector of model **A.1** —%sheet, %helix, %longest sheet, %longest helix—was used to develop a model separately for each of the other four virus families. For each of these models the %correctly classified ranged from 96.2% to 100% with a sensitivity ranging from 0.86 to 1.0, and a specificity ranging from 0.98 to 1.0. To place these results in context, the class-balanced scores for these datasets ranged from 86.8% to 88.5%. For three of the four classifiers, there was a statistically significant difference between class-balanced scores and model accuracy, with *p*-values ranging from 0.004 to 0.039; the exception being the Paramyxoviridae classifier which gave a *p*-value of 0.056.(two-tailed Fisher’s Exact Test) (see S1 Table). Models based on combining total N-sites, density, %sheet, %helix, and %longest helix were also generated for each virus family (as for **B.1**), respectively; in this case, the % correctly classified ranged from 93.5% to 100% (compared with class-balanced scores from 86.5% to 87.5%), and two-tailed Fisher’s exact test *p*-values ranging from 0.004 to 0.238.

These two-feature vectors (associated with the A.1 and B.1 models, respectively) were then used to create cross-respiratory-virus family models which were validated by applying 10-fold cross validation over the full five-family training set. Models **A** and **B** yielded %correctly classified of 97.32% and 97.03%, respectively, relative to a class-balanced score of 87.66%, and had an AUC for the ROC curve of 0.977 and 0.985, respectively (see Table 4).

**Table 4:** ML models ability to differentiate spike from non-spike for the five families.

Model	Features	Class Balance	Correctly Classified	P-value	AUC/CI (95%)
A	%sheet, %helix, %longest sheet, %longest helix	87.66%	97.32%	0.000003	0.977 ± 0.014
B	total N-sites, density, %sheet, %helix, %longest helix	87.66%	97.03%	0.000007	0.985 ± 0.014

Further model assessment was performed on the extra-familial validation set, a set consisting of cytomegaloviruses and alpha herpesviruses with known respiratory activity [41] from a sixth viral family (Herpesviridae) to which the model was naïve. For this extra-familial set with a class-balanced score of 83.33%, model **A** yielded a %correctly classified of 95.83% with an AUC of 1, whereas model **B** yielded a %correctly classified of 83.33% with an AUC of 0.9 (see Table 5). Cross-virus family models **A** and **B** are described in detail in Table 4.

**Table 5:** ML models ability to differentiate spike from non-spike on extra-familial set.

Model	Features	Class Balance	Correctly Classified	P-value	AUC/CI (95%)
A	%sheet, %helix, %longest sheet, %longest helix	83.33%	95.83%	0.01	1.00 ± 0.13
B	total N-sites, density, %sheet, %helix, %longest helix	83.33%	83.33%	0.3	0.91 ± 0.13

As a further check against class imbalance, a 50–50 balanced bootstrapping set was generated. Model **A** and **B** were then trained on this balanced dataset and their performance on the extra-familial validation set was compared to the corresponding performance of the non-bootstrapped model on the same set. For both Models **A** and **B**, the %correctly classified by the bootstrapped 50–50 balanced model was identical to that of non-bootstrapped model, with 95.83% for Model **A** and 83.33% for Model **B** (see Table 6). Upon visual inspection of the random forests of both non-bootstrapped and bootstrapped models, strong similarities in tree structure and values for decision nodes were observed suggesting that bootstrapping did not change the signal captured during training, leading to the identical performance on the extra-familial validation set.

**Table 6:** Bootstrapped class-balanced models performance on extra-familial set.

Model	Features	Class Balance	Correctly Classified	P-value	AUC/CI (95%)
Bootstrap A	%sheet, %helix, %longest sheet, %longest helix	83.33%	95.83%	0.01	1.00 ± 0.13
Bootstrap B	total N-sites, density, %sheet, %helix, %longest helix	83.33%	83.33%	0.3	0.89 ± 0.13

## Discussion

It has previously been shown, prior to the emergence of SARS-CoV-2, that across coronaviruses the tertiary structure of the spike protein is not conserved although the connectivity of secondary structure elements is [8]. As evidenced in Fig 1A, the tertiary structure of the “spike” protein is clearly not conserved across different respiratory families. The pattern of N-linked glycosylation of the spike protein is, however, conserved and may play a role in immune evasion [9, 50]. Given these insights, we set out to explore whether ML models based on predicted secondary elements alone or in combination with predicted N-glycosylation sites could be developed to classify “spike” vs. non-spike from a sequence of an unknown respiratory virus.

Model **A** (based on predicted secondary structure elements alone) and model **B** (based on that plus predicted N-glycosylation sites) perform well on the five respiratory virus family training set with accuracies just over 97 and low bias errors as shown by 10-fold cross validation. This result is particularly noteworthy given that the coronaviruses in the feature selection set were human as were all the other viral sequences in the training set, while the coronaviruses in the training set are from animal species. On the herpes extra-familial validation set the performance of model A was maintained (96% correctly classified) while that for model B was not (83%, the same value as the class-balanced score). The extra-familial validation set is a particularly difficult test of the models in that Herpesviridae viruses have roughly 10 glycoproteins that are not immediately responsible for cell entry, that instead act to activate the primary fusogenic glycoprotein or facilitate transport of proteins between the Golgi network and the membrane [51]. These additional glycoproteins may lead to false positives that worsen model accuracy. These data taken together point to the robustness of each cross-family model for the five major respiratory families the model was trained on but suggest that only model **A** may be fully robust when considering a new viral family.

Our model has limitations in that it was trained on a non-balanced set. This non-balanced nature of “spike” vs. non-spike in the original sets, however, is reflective of the true distribution in nature. In addition, bootstrapped models were also generated from the training set by utilizing datasets that were 50–50 balanced for “spike” vs. non-spike; this was done to eliminate the possibility that the accuracy of the models could be due to the fact that non-spike was overrepresented in the sets. Irrespective of class balance, models **A** and **B** performed equally well at differentiating “spike” from non-spike for respiratory virus sequences. Another potential weakness of our analysis is that the extra-familial validation test set may be too stringent in that while Herpes viruses can cause respiratory symptoms they are not generally thought of as respiratory family viruses. Furthermore, since the model was trained on viruses that elicit respiratory illness, its utility on viral sequences in general is unknown.

These models could be useful in the pre-pandemic stages of an emerging respiratory pathogen, aiding in a rapid response to prevent to prevent outbreaks from growing into a pandemic. Allowing researchers to identify the viral surface glycoprotein responsible for host cell entry within seconds with a high degree of confidence for an unknown viral sequence, would provide the global community with the opportunity to quickly focus on a key drug and vaccine target. The models could also help to characterize pathogens of concern (as, e.g., the WHO priority diseases 2022 list) prior to the epidemic stage, aiding in preparedness.

Beyond the predictive power of the models, perhaps the most-interesting finding of this work relates to the signal in the data, suggesting that surface proteins can be characterized by their secondary structure elements and sequence prevalence. Like the SARS-CoV-2 spike proteins, most viral surface glycoproteins responsible for host cell entry have minimal extended sheets relative to helices. The helices tend to run anti-parallel in the pre-fusion protein, and undergo a conformational change to a form with longer contiguous helices post-fusion [52]. Proteins with higher %helix and %longest helix may be more likely to undergo this conformational change. The likelihood may be further increased if the protein has a low %sheet and %longest sheet.

The relationship between structure and function has long been discussed at the tertiary level [53–56]. This work points to a relationship that is discernable and meaningful even at the secondary structure level through ML approaches. Furthermore, this signal in the data can be captured by using standard methods to predict the secondary structural elements from sequence alone. Taken together this work suggests that models of these types based on predicted secondary elements and sequence prevalence could potentially be further developed in the future for rapid sequence annotation in general.

## Supporting information

S1 Table All ML models examined. (PDF)

S1 Fig Calculation of secondary structure elements. A flowchart showing the process for an automated script calculating the secondary structure elements with Jpred4.
(PDF)

S1 Dataset ARFF data for models based on secondary structure. (ARFF)

S2 Dataset ARFF data for models based on secondary structure and N-glycosylation sites. (ARFF)

S3 Dataset ARFF data for extra-familial dataset for models based on secondary structure. (ARFF)

S4 Dataset ARFF data for extra-familial dataset for models based on secondary structure and N-glycosylation sites. (ARFF)
