# Supplementary material for: Machine learning for the identification of respiratory viral attachment machinery from sequences data
Source: PLoS One. 2023 Mar 2;18(3):e0281642. doi: 10.1371/journal.pone.0281642 (PMC9980812; doi:10.1371/journal.pone.0281642)
Supplement: S1 Table — (PDF) [file pone.0281642.s001.pdf]

S1 Table. All ML Models Examined.

Model #	Viral Family	Features	Class Balance Score	Correctly Classified	Fisher's Exact Test
1, **C.1**	Coronaviridae	total N-sites, density, %N, %M, %S	86.4%	98.648%	0.028
2	Coronaviridae	total N-sites, density, %M, %S	86.4%	98.648%	0.028
3	Coronaviridae	total N-sites, density	86.4%	98.648%	0.028
4, **D.1**	Coronaviridae	total N-sites, density, %M, %S, %sheet, %helix, %longest sheet, %longest helix	86.4%	98.648%	0.028
5, **B.1**	Coronaviridae	total N-sites, density, %sheet, %helix, %longest helix	86.4%	98.648%	0.028
6, **A.1**	Coronaviridae	%sheet, %helix, %longest sheet, %longest helix	86.4%	98.648%	0.028
7, **A.1**	Paramyxoviridae	%sheet, %helix, %longest sheet, %longest helix	87.097%	100.000%	0.056
8, **A.1**	Pneumoviridae	%sheet, %helix, %longest sheet, %longest helix	88.462%	98.077%	0.004
9, **A.1**	Adenorividae	%sheet, %helix, %longest sheet, %longest helix	87.500%	98.438%	0.015
10, **A.1**	Orthomyxoviridae	%sheet, %helix, %longest sheet, %longest helix	87.500%	96.875	0.039
11, **B.1**	Paramyxoviridae	total N-sites, density, %sheet, %helix, %longest helix	87.097%	93.548%	0.238
12, **B.1**	Pneumoviridae	total N-sites, density, %sheet, %helix, %longest helix	86.462%	98.077%	0.004
13, **B.1**	Adenorividae	total N-sites, density, %sheet, %helix, %longest helix	87.500%	98.438%	0.015
14, **B.1**	Orthomyxoviridae	total N-sites, density, %sheet, %helix, %longest helix	87.500%	96.875%	0.039
15, **C**	Coronaviridae + Paramyxoviridae + Pneumoviridae + Adenorividae + Orthomyxoviridae	total N-sites, density, %N, %M, %S	87.658%	92.721%	0.011
16, **D**	Coronaviridae + Paramyxoviridae + Pneumoviridae + Adenorividae + Orthomyxoviridae	total N-sites, density, %M, %S, %sheet, %helix, %longest sheet, %longest helix	87.658%	98.101%	<0.001

17, **A**	Coronaviridae + Paramyxoviridae + Pneumoviridae + Adenorividae + Orthomyxoviridae	%sheet, %helix, %longest sheet, %longest helix	87.658%	97.785%	<0.001
18, **B**	Coronaviridae + Paramyxoviridae + Pneumoviridae + Adenorividae + Orthomyxoviridae	total N-sites, density, %sheet, %helix, %longest helix	87.658%	98.101%	<0.001
