# Supplementary material for: Machine learning for the identification of respiratory viral attachment machinery from sequences data
Source: PLoS One. 2023 Mar 2;18(3):e0281642. doi: 10.1371/journal.pone.0281642 (PMC9980812; doi:10.1371/journal.pone.0281642)
Supplement: S1 Fig — A flowchart showing the process for an automated script calculating the secondary structure elements with Jpred4. (PDF) [file pone.0281642.s002.pdf]

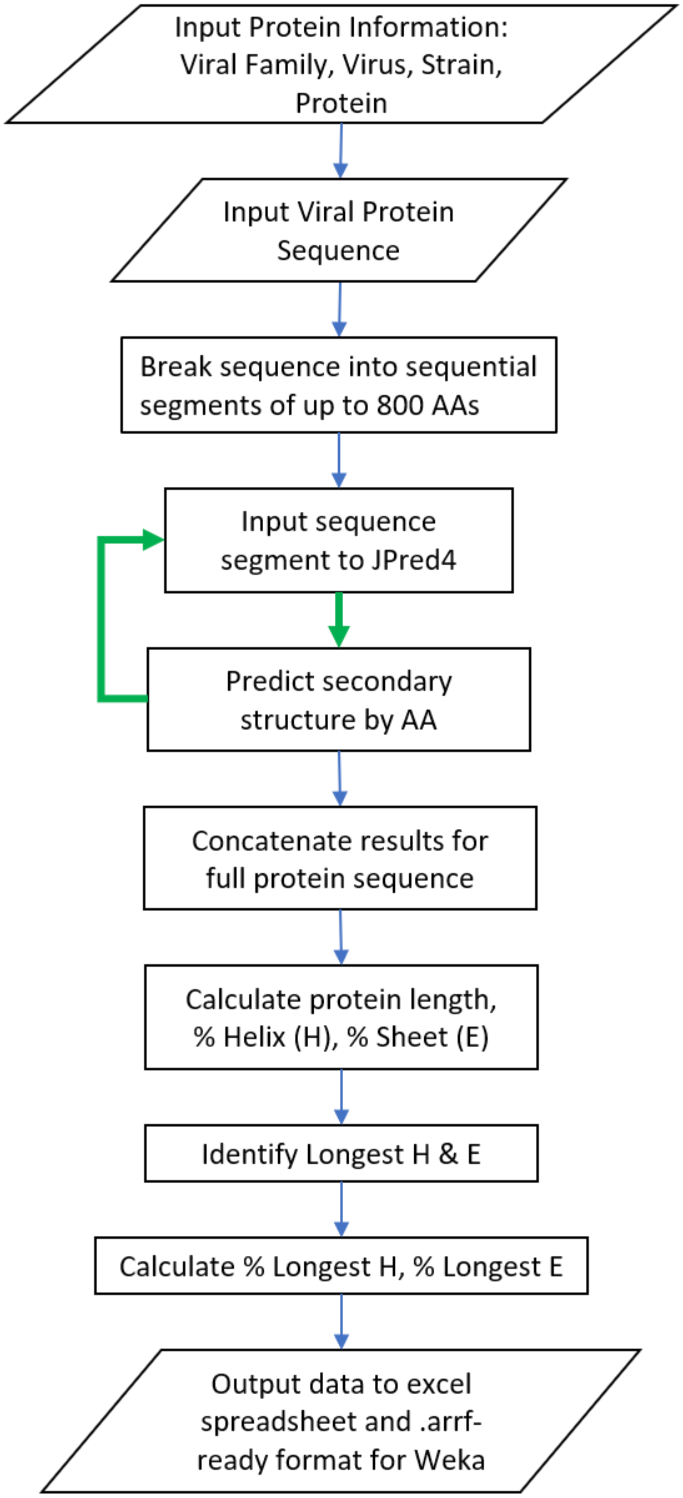

S1 Fig. Calculation of secondary structure elements. A flowchart showing the process for an automated script calculating the secondary structure elements with Jpred4.
